# Prevalence and factors associated with higher levels of perceived stigma among people with schizophrenia Addis Ababa, Ethiopia

**DOI:** 10.1186/s13033-020-00348-9

**Published:** 2020-03-13

**Authors:** Getachew Tesfaw, Boki Kibru, Getinet Ayano

**Affiliations:** 1 Department of Psychiatry, College of Medicine and Health Science, University of Gondar, Gondar, Ethiopia; 2 Gefersa Mental Health Rehabilitation Centre, Addis Ababa, Ethiopia; 3 Research and Training Department, Amanuel Mental Specialized Hospital, Addis Ababa, Ethiopia; 4 School of Public Health, Curtin University, Perth, Australia

**Keywords:** Perceived stigma, Schizophrenia, Factors

## Abstract

**Background:**

Schizophrenia is a severe and disabling chronic mental disorder and accompanied by different levels of a perceived stigma that affects almost all age groups. This perceived stigma negatively impacts the quality of life, physical, and mental well-being of people with schizophrenia. It is also linked with a poor level of functioning, poor adherence to drugs, and increased dropout rate. However, research into perceived stigma and associated factors among people with schizophrenia in low- and middle-income countries are limited. Therefore, this study aimed to explore the perceived stigma and correlates among people with schizophrenia in Ethiopia.

**Methods:**

An institution based cross-sectional study was conducted from May to June 2018. A structured, pre-tested, and interviewer-administered questionnaire was used to collect data. The standardized perceived devaluation and discrimination questionnaire was used to assess perceived stigma. The systematic random sampling technique was used to select study participants. Binary logistic regression analysis was used to identify factors associated with perceived stigma. An odds ratio (OR) with a 95% confidence interval (CI) was computed to assess the strength of the association.

**Results:**

The prevalence of high perceived stigma was found to be 62.6% [95% CI 58.3, 67.4]. In the multivariate logistic regression, female sex [AOR = 2.30, 95% CI 1.42, 3.71], age of onset of schizophrenia [AOR = 1.85, 95% CI 1.19, 2.89], multiple hospitalizations [AOR = 1.7, 95% CI 1.16, 3.27], and duration of illness 1–5 years [AOR = 2, 95% CI 1.01, 3.27], 6–10 years [AOR = 2.48, 95% CI 1.29, 4.74], and ˃10 years [AOR = 2.85, 95% CI 1.40, 5.79] were factors significantly associated with higher perceived stigma.

**Conclusion:**

In the present study, the prevalence of high perceived stigma among people with schizophrenia was found to be 62.6%. Female sex, age of onset of schizophrenia, multiple hospitalizations, and duration of illness were factors significantly associated with higher perceived stigma. Measures to enhance the awareness of the patients, their families and their social networks about perceived stigma and associated factors, by the leading government and healthcare institutions are warranted.

## Introduction

Stigma refers to negative beliefs and attitudes that lead people to reject, avoid, or fear and those perceive as being different [1, 2]. Perceived stigma (PS) is the fear of being discriminated against or the fear of enacted stigma, which arises from society’s belief [3]. Stigmatized persons may internalize perceived prejudices and develop negative feelings about themselves and patients feel shame and embarrassment about having the mental illness [4]. Stigmatized patients are unwilling to seek services, social interaction, and employment opportunities [5, 6].

The global magnitude of perceived stigma among people with schizophrenia from 16 countries was found to be 13.5% and 22.1% for developing countries and 11.7% in developed ones, respectively [7]. In another study conducted in 14 European countries, the prevalence of stigma was 41.7% among schizophrenia patients [8]. In Ethiopia, the prevalence of stigma among people with mental illness were reported to be within the range of 32.1% to 83.5% [9, 10].

People with mental illness are subjected to high levels of stigma and discrimination because of widely held misconceptions about the causes and nature of mental health situations [11]. The stigma of mental illness is a severe burden for people with mental illness in both their private and public lives, and it also affects their relatives [12]. The causes and nature of mental illness have been attributed to supernatural forces, and praying has been the treatment of choice to deal with the problem [13, 14]. No one has the right to avoid mentally ill persons, but doctors and psychiatric nurses in psychiatric hospitals have a negative view of the people living with schizophrenia and their families [13, 15, 16].

Globally, people with mental disorders are victimized for their illnesses and unfair discrimination in accessing housing, jobs, and normal community opportunities [17]. Mental health related stigma leads to avoidance of labeling oneself negatively and it is negatively associated with quality of life, functioning, treatment avoidance, an increased dropout rate, reduced adherence, homelessness, suicide, and other positive health outcomes [1, 18–20].

Different epidemiologic studies suggest that perceived stigma is highly prevalent among patients with schizophrenia and is influenced by the type and severity of psychopathology, coping skills, insight, casual beliefs, social support, and self-esteem [21, 22]. According to scientific evidence stigma among the mentally ill (including schizophrenia) is associated with a range of negative emotional and psychological impacts such as diminished self-efficacy [23], low self-esteem [23, 24], and depressive symptoms [23].

A number of factors have been found to be significant predictors of perceived stigma among patients with schizophrenia from previous studies including educational status, residency, social withdrawal, non-adherence to medication, duration of illness, marital status, old age, and low self-esteem [9, 13, 18, 25–27].

Moreover, perceived stigma negatively impacts the quality of life, physical, and mental well-being of people with schizophrenia [21, 28–31]. It is also linked with a poor level of functioning, poor adherence to drugs, and an increased dropout rate [9, 32]. However, research into perceived stigma and associated factors among people with schizophrenia in low- and middle-income countries are limited. Therefore, this study aimed to explore the perceived stigma and correlates among people with schizophrenia in Ethiopia.

## Methods

An institution-based cross-sectional study was conducted from May to June 2018 at Amanuel Mental Specialized hospital Addis Ababa, Ethiopia. It is the only mental health hospital in the country with 300 beds and 3420 schizophrenia patients on monthly follow-ups. All patients with schizophrenia included in the sample were on follow-ups at Amanuel Mental Specialized hospital in the outpatient department, while patients found to be severely ill (incoherent and actively psychotic) were excluded from the study.

### Sample size calculation and sampling technique

The sample size was calculated by using a single population proportion formula involving the using Epi-info version 7 with a 95% CI, a 5% margin of error and taking the prevalence of stigma 46.7% from a previously published study in our country [18]. This yields a total of 384 samples. Having assumed 10% a non-respondent rate, the final sample size was 384 + 39 = 423 schizophrenic patients were recruited randomly by using the systematic sampling technique.

### Measurements

Data were collected using a pre-tested interviewer-administered questionnaire, which contained several other explanatory variables include socio-demographic characteristics (age, sex, education, marital status and others), clinical factors (duration of illness, suicidal ideation, suicide attempts, number of hospitalizations, negative, positive, and general psychopathology symptoms of schizophrenia), social support, and substance use (alcohol, cigarette, and chat).

Social support was assessed by using the Oslo 3-item social support scale and used several studies. It provides a brief measure of social support and functioning and is considered to be one of the best predictors of mental health. It covered different levels of social support by measuring the number of people the respondents feel close to, the interest and concern showed by others and the ease of obtaining practical help from others. In order to OSS-3, total scores are calculated by adding up the raw scores for each item. The sum score scale ranges from 3 to 14 and has three broad categories: “Poor social support” 3–8, “Moderate support” 9–11, and “Strong support” 12–14 (the reference on social support), respectively [33]. It has been used in Ethiopia in various settings, including the community [34–36]. Suicidal ideation and attempts measured according to the WHO questionnaire. If a respondent provided a “Yes” answer to the question, “Have you ever seriously thought about committing or attempted suicide, respectively? They were considered having suicide, respectively [37]. The positive and negative syndrome scale (PANSS) is a well-validated tool for measuring psychopathology in patients diagnosed with psychotic disorders and has 30 questions consisting of three subscales; seven positives, seven negatives, and sixteen general psychopathology questions [38]. It was used in schizophrenia patients in the previous study in Ethiopia [39].

In this study, perceived stigma was measured by using a 12-items perceived devaluation and discrimination (PDD) scale questionnaire. It was used to measure the extent to which a person believes that most people were devaluated and discriminated against someone with psychiatric problems [40]. Each item of PDD is rated on a 4-point Likert scale which ranged from strongly disagree to strongly agree (1 = strongly disagree, 2 = disagree, 3 = agree, and 4 = strongly agree). A high level of perceived devaluation and discrimination is indicated by agreement with six of the items and disagreements with six others and possible scores ranges from 1 to 4 so that a higher score showed a higher level of perceived stigma, but items 1, 2, 3, 4, 8, and 10 were reverse direction [24, 41]. The prevalence of high perceived stigma defined as an item mean score of 2.5 or a higher on the mean aggregated scale score represents the midpoint 1 to 4 item scale on PDD scales. Then perceived stigma scores have dichotomized those participants scoring greater than or equal to the mean score of 2.5 on PDD scales as having “high perceived stigma” and those scoring below the mean value considered as having “low perceived stigma” [42]. It is widely used in different settings in different countries including Ethiopia [8, 9, 13].

### Data analysis

Data were entered into Epi-info software after checking the completeness and transferred to SPSS version 21 for analysis. Binary and multivariable logistic regression analyses were done to see the association of each independent variable with the outcome variable. The strength of the association was evaluated using the adjusted odds ratio with a 95% CI, and a P-value of less than 0.05 was considered statistically significant.

## Results

### Socio-demographic characteristics

From a total of 423 participants invited to participate in this study, 409 completed the survey with a response rate of 96.7%. The mean age of the respondents was 22 (± 9.73) years. Out of the participants, over two-thirds (62.3%) were male and 242 (59.2%) were single. Almost half (50.95%) of the participants were Orthodox Christian, and 170 (41.6%) had a primary level of education (Table 1).

**Table 1:** Socio-demographic characteristics among schizophrenia patients at AMSH, Addis Ababa, Ethiopia, 2018 (N = 409)

Variables	Categories	Frequency	Percent
Age	18–25	154	37.6
	26–35	132	32.3
	36–45	81	19.8
	≥ 46	42	10.3
Sex	Male	255	62.3
	Female	154	37.7
Religion	Orthodox	208	50.9
	Muslim	141	34.5
	Protestant	60	14.6
Marital status	Single	242	59.2
	Married	167	40.8
Ethnicity	Oromo	165	40.4
	Amhara	137	33.6
	Gurage	82	20
	Tigre	25	6
Educational status	Unable to read and write	41	10
	Primary education	170	41.6
	Secondary education	139	34
	Diploma and above	59	14.4

### Clinical characteristics

Of the 409 participants, 215 (53%) had age onsets of schizophrenia after 25 years; one-third (31%) of the respondents had 6–10 years duration of illness and nearly one-third (28.1%) had less than or equal to two admissions. In this study, a considerable proportion of people with schizophrenia had a history of suicide attempts 210 (51.3%). The mean and standard deviation of positive, negative and general psychopathology symptoms were 11 ± 3.9, 9.4 ± 3, and 23.92 ± 5.4, respectively as measured by using PANAS (Table 2).

**Table 2:** Distribution of clinical characteristics among Schizophrenia Patients at AMSH, Addis Ababa, Ethiopia, 2018 (N = 409)

Variables	Categories	Frequency	Percent
Age of onset of Schizophrenia	Before 25 years	192	47
	After 25 years	215	53
Duration of illness (years)	< 1	75	18
	1–5	117	29
	6–10	127	31
	> 10	90	22
Number of admissions	No	238	58.2
	≤ 2	115	28.1
	> 2	56	13.7
Suicidal ideation	Yes	202	49.4
	No	207	50.6
Suicide attempts	Yes	210	51.3
	No	199	48.7
Positive symptoms	Mean and SD	11.8 ± 5	–
Negative symptoms	Mean and SD	9.4 ± 3	–
Pathology of symptoms	Mean and SD	23.92 ± 5.4	–

### Social and substance-related factors

Regarding social factors, 46.7% and 45.5% of the participants had moderate and poor social supports, respectively. At the moment, 261 (63.8%) were smoking tobacco; 236 (57.7%) were taking alcohol in their lifetime, and about two-thirds of the participants (59.2%) were chewing chat in their lifetime (Table 3).

**Table 3:** social and substance use characteristics among schizophrenia patients at AMSH, Addis Ababa, Ethiopia, 2018 (N = 409)

Variables	Categories	Frequency	Percent
Social support	Poor	186	45.5
	Medium	191	46.7
	Good	32	7.8
Current use			
Tobacco	Yes	261	63.8
	No	148	36.2
Alcohol	Yes	236	57.7
	No	173	42.3
Khat (chat)	Yes	244	59.7
	No	165	40.3
Lifetime use			
Tobacco	Yes	325	79.5
	No	84	20.5
Alcohol	Yes	321	78.5
	No	88	21.5
Khat (chat)	Yes	334	81.7
	No	75	18.3

### Prevalence of perceived stigma

This study revealed that the prevalence of high perceived stigma among people with schizophrenia was 62.6%, with a 95% CI (58.3, 67.4) (Table 5). Regarding frequency and percentage of high perceived stigma toward each item, 286 (69.9%) agreed with the item “Most employers will pass over the application someone who has had illness in favor of another applicant”, 27 (6.6%) strongly agreed with the item “Most people would willingly accept a person who has had mental illness as a close friend”, 269 (65.8%) were disagreed with the item “Once they know a person was in a mental hospital for treatment, most people will lake his/her opinions less seriously”, and 10 (2.4%) were strongly disagreed to item “Most people believe that entering a mental hospital is a sign of personal failure” (Table 4).

**Table 4:** Distribution of participants by their response to perceived stigma of mental illness scale at AMSH, Addis Ababa, Ethiopia, 2018 (N = 409)

No	The 12 items of perceived devaluation and discrimination (PDD) scale	Strongly disagree	Disagree	Agree	Strongly agree
1	Most people would willingly accept a person who has had mental illness as a close friend	3 (0.7%)	226 (55.3%)	153 (37.4%)	27 (6.6%)
2	Most people believe that a person who has been hospitalized for mental illness is just as intelligent as the average person	2 (0.5%)	290 (70.9%)	112 (27.4%)	5 (1.2%)
3	Most people believe that a person who has had mental illness is just as trustworthy as the average citizen	1 (0.2%)	229 (56%)	162 (39.6%)	17 (4.2%)
4	Most people would accept a person who has fully recovered from mental illness as a teacher of young children in a public school	3 (0.7%)	188 (46%)	207 (50.6%)	11 (2.7%)
5	Most people believe that entering a mental hospital is a sign of personal failure	10 (2.4%)	222 (54.3%)	174 (42.5%)	3 (0.7%)
6	Most people will not hire a person who has had a mental illness to take care of their children, even if he or she had been well for some time	10 (2.4%)	203 (49.6%)	195 (47.7%)	1 (0.2%)
7	Most people think less of a person who has been in a mental hospital to treat	5 (1.2%)	193 (47.2%)	206 (50.4%)	5 (1.2%)
8	Most employers will hire a person who has had mental illness if he or she is qualified for the job	8 (2.0%)	277 (67.7)	122 (29.8)	2 (0.5%)
9	Most employers will pass over the application someone who has had mental illness in favor of another applicant	2 (0.5%)	113 (27.6)	286 (69.9)	8 (2.0%)
10	Most people in my community would treat someone who has had mental illness just as they would treat anyone	2 (0.5%)	248 (60.6%)	153 (37.4%)	6 (1.5%)
11	Most young women would be reluctant to date a man who has been hospitalized for a serious mental illness	5 (1.2%)	258 (34.1%)	142 (34.7%)	4 (1.0%)
12	Once they know a person was in a mental hospital for treatment, most people will take his/her opinions less seriously	6 (1.5%)	269 (65.8%)	129 (31.5%)	5 (1.2%)

### Factors associated with high perceived stigma

Among all explanatory variables, those variables with P-values less than 0.2 in the bi-variable logistic regression were included in the multivariable logistic regression model. Additionally, we considered Bradford Hill’s causal criteria, including the consistency of the association [43]. These variables included sex, marital status, age of onset of schizophrenia, numbers of hospitalizations, duration of illness, and current tobacco. The model goodness of fit checked by using the Hosmer and Lemeshow test and a P-value was 0.65. So, the model was good. The multivariate analyses suggest that female sex was 2.4 times more likely [AOR = 2.4, 95% CI 1.42, 3.71] to have higher perceived stigma compared to males. Age of onset of schizophrenia before 25 years was a 1.85 times [AOR = 1.85, 95% CI 1.19, 2.89] more likely to develop higher perceived stigma compared to counterparts. Furthermore, the odds of higher perceived stigma increased by 1.7 times [AOR = 1.7, 95% CI 1.16, 3.27] for respondents who had one or two numbers of hospitalization compared to no admission. Moreover, significant association had between higher perceived stigma and those participants with the duration of the illness for 1–5 years [AOR = 2:00, 95% CI 1.01, 3, 27], 6–10 years [AOR = 2.48, 95% CI 1.29, 4.74], and ˃ 10 years [AOR 2.85, 95% CI 1.40, 5.79] compared to patients’ illness less than one year **(**Table 5).

**Table 5:** Bi-variable and multivariate analysis of stigma among schizophrenia patients at AMSH, Addis Ababa, Ethiopia, 2018 (n = 409)

Variables	Categories	Stigma		COR (95% CI)	AOR (95% CI)	P-Value
		Low	High			
Sex	Male	117	138	1:00	1:00	
	Female	36	118	2.80 (1.80, 4.34)	2.30 (1.42, 3.71)*	*0.04*
Marital status	Single	101	141	0.63 (0.41, 0.96)	0.64 (0.41,1.01)	0.31
	Married	52	115	1:00	1:00	
Age of onset of schizophrenia	Before 25 years	57	137	1.94 (1.29, 2.92)	1.85 (1.19, 2.89)**	*0.01*
	After 25 years	96	119	1:00	1:00	
Number of admissions	Never	100	138	1:00	1:00	
	≤ 2	33	82	1.80 (1.11, 2.90)	1.94 (1.16, 3.27)*	*0.01*
	> 2	20	36	1.30 (0.71, 2.39)	1.70 (0.83, 3.39)	0.32
Duration of illness (years)	< 1	45	30	1:00	1:00	
	1–5	45	72	2.4 (1.32, 4.34)	2.00 (1.01, 3.27)**	*0.01*
	6–10	38	89	3.513 (1.932, 6.389)	2.48 (1.29, 4.74)***	*< 0.001*
	> 10	25	65	3.9 (2.03, 7.492)	2.85 (1.40, 5.79)***	*< 0.001*
Current Tobacco use	Yes	87	174	1.61 (1.06, 2.435)	1.36 (0.84, 2.18)	0.23
	No	66	82	1:00	1:00	

Italic values indicate significance of p-value (P < 0.05)

* P<0.05; ** P<0.01; *** P<0.001

## Discussion

In the current study, the prevalence of higher perceived stigma and its possible association with various factors was assessed. The results revealed that a significant proportion of people with schizophrenia had a higher perceived stigma. The prevalence of high perceived stigma among people with schizophrenia was found to be 62.6%. This result is consistent with the reported magnitude of high perceived stigma in studies carried out among schizophrenia and schizoaffective disorder patients in Austria 66.9% [44], a meta-analysis report of the magnitude perceived stigma among schizophrenia patients in the USA, which revealed a rate of 64.55% [22], and the magnitude of self-stigma among schizophrenia spectrum disorders in Czech-Republic was 63.32% [45].

However, this finding was lower than that in the study conducted in Ethiopia, where prevalence of higher perceived stigma was reported at 83.5% before 6 years back by using the perceived devaluation and discrimination scale and in this study, factors associated with perceived stigma completely different from the previous study [9]. Another study conducted in southern California showed that 80% of people with schizophrenia had experienced perceived stigma [46]. Similarly, in 2008 study conducted in Australia also revealed that nearly three out of four (74%) of people with schizophrenia had experienced perceived stigma [47]. The reason for the variation of the above prevalence might be due to the sample size differences, the different years where studies were carried out, the use of various scales for assessing the levels of higher perceived stigma, the level of awareness about mental illness across the countries, and socio-cultural differences between across the countries.

On the other hand, the prevalence of the current study is higher than that in studies done in two areas of Ethiopia and Taiwan, in which the prevalences were estimated to be 46.7%, 32.1%, and 39%, respectively [10, 18, 48]. The discrepancy of the above rate of prevalence might be because of types of stigma difference, measurement tool variations, time differences, study population differences, sample size variations, study designs, and perhaps because of the socio-cultural distinctions between Ethiopia and Taiwan.

Female patients were 2.4 times greater risk for high perceived stigma than male patients, which was in line with two studies done in Ethiopia and India [10, 26, 49]. Another study done in Ethiopia reported significantly higher self-stigma levels in females than males [50]. Similarly, perceived stigma among people with schizophrenia in 14 European countries showed higher levels of self-stigma in females [8]. This may be due to the fact that the studies were carried out in different years; various scales were used for assessing the levels of perceived stigma, and that different country with various socio-cultural backgrounds. For example, in Ethiopia female sex experiencing some form of trauma during their lives like gender violence, discrimination, and mistreatment of women associated with mental illness leading to high stress, social withdrawal, and low self-esteem.

Those with an age of onset of schizophrenia before 25 years had a 1.85 times greater likelihood of developing higher perceived stigma as compared to those patients with age of onset above 25 years. This means that the patients with early onset of schizophrenia feel that they are stigmatized more often than those patients with late-onset. This was consistent with the results from studies conducted in the Netherland and the Czech Republic [28, 51], and India [52]. The possible reason could be the early onset of schizophrenia may impair the development of personality and the patient’s social roles before the patient can learn how to manage these situations.

A history of being hospitalized once or twice increased higher perceived stigma by 1.94 times among schizophrenia patients when compared to patients who did not have a history of admission. This is in line with the findings of other studies done in two areas of the Czech Republic [45, 51]. The number of previous admissions presented high vulnerability to higher perceived stigma among schizophrenia patients. The reason for higher odds of developing perceived stigma in those patients with higher rates of admission (1 or 2) might be due to those who were hospitalized might be unfairly discriminated against by health professionals due to their behavior as well as negative attitudes of the community after the patients returned to their homes when they are discharged from the hospital. Another possible explanation for the association between perceived stigma and the number of hospitalizations maybe due to those admitted patients who had more likely with a chronic and severe illness which had higher rates of perceived stigma compared with less severe stages of the illness which requires no hospitalizations.

Furthermore, the duration of illness greater than 1 year had higher odds of having high perceived stigma compared to those patients with the duration of the illness less than 1 year. This is in line with the findings of the other two studies that were done in Ethiopia and the Czech Republic. Self-stigma was highly associated with an extended duration of illness among schizophrenia patients [45]. In a similar study done in India among hospitalized schizophrenia patients, the mean duration of illness of 12.6 years was significantly correlated with high perceived stigma [52]. This might be because of the long duration of illness that might lead to negative symptoms of schizophrenia, in particular, cognitive distortions, and social withdrawal may increase perception of self-stigmatization.

### Strength and limitations of the study

The present study has several strengths. Firstly, we used standardized instruments to measure perceived stigma; secondly, we adjusted the final multivariable model for most important factors affecting stigma, including the age of onset of schizophrenia, substance use, number of hospital admissions, as well as the duration of the illness.

This study also has some limitations; first, it was a cross-sectional study and thus cannot establish a causal link between associated factors and perceived stigma among schizophrenic patients; second, since the participants were schizophrenic, recall bias might underestimate the magnitude due cognitive impacts of the disorder.

## Conclusion

In the present study, the prevalence of high perceived stigma among people with schizophrenia was found to be 62.6%. Female sex, age at onset of schizophrenia, having multiple hospitalizations, and duration of illness greater than 1 year were significantly associated with higher perceived stigma. Measures to enhance the awareness of the patients, their families and their social networks about high perceived stigma by the leading government as well as healthcare institutions are warranted.

## Data Availability

The dataset during and/or analyzed during the current study available from the corresponding author on reasonable request.

## Abbreviations

AMSH: Amanuel Mental Specialized Hospital
AOR: Adjusted odds ratio
DSM-V: Diagnostic statistical manual of fifth edition text Revision
ISMI: Internalized Stigma of Mental Illness
SPSS: Statistical Package for Social Science
OR: Odds ratio
UoG: University of Gondar
USA: United States of America
WHO: World Health Organization
